# Regenerative Approaches and Future Trends for the Treatment of Corneal Burn Injuries

**DOI:** 10.3390/jcm10020317

**Published:** 2021-01-16

**Authors:** Gabriele Saccu, Valeria Menchise, Cristina Giordano, Daniela Delli Castelli, Walter Dastrù, Rinaldo Pellicano, Emanuela Tolosano, Phuc Van Pham, Fiorella Altruda, Sharmila Fagoonee

**Affiliations:** 1 Department of Molecular Biotechnology and Health Sciences, Molecular Biotechnology Center, University of Turin, 10126 Turin, Italy; gabrisaccu@gmail.com (G.S.); daniela.dellicastelli@unito.it (D.D.C.); walter.dastru@unito.it (W.D.); emanuela.tolosano@unito.it (E.T.); 2 Institute of Biostructure and Bioimaging, National Research Council, Molecular Biotechnology Center, 10126 Turin, Italy; 3 Ophthalmology Veterinary Practice, c.so Galileo Ferraris 121, 10126 Turin, Italy; cristina.giordano@unito.it; 4 Unit of Gastroenterology, Molinette Hospital, 10126 Turin, Italy; rinaldo_pellican@hotmail.com; 5 Laboratory of Stem Cell Research and Application, and Stem Cell Institute, VNUHCM University of Science, Ho Chi Minh City 08000, Vietnam; pvphuc@hcmuns.edu.vn

**Keywords:** cornea, stem cells, extracellular vesicles, burn injury, wound healing, imaging

## Abstract

Ocular chemical and thermal burns are frequent causes of hospitalization and require immediate interventions and care. Various surgical and pharmacological treatment strategies are employed according to damage severity. Controlling inflammation and neovascularization while promoting normal ocular surface anatomy and function restoration is the principal aim. In the most severe cases, when epithelial healing is severely affected, reconstruction of the ocular surface may be a valid option, which, however, requires expertise, adequate instruments, and qualified donors. Numerous endogenous and exogenous strategies have been considered for corneal repair. Among these, stem cells and their derivatives have offered numerous attractive possibilities in finding an effective way in stimulating corneal regeneration. Limbal epithelial stem cells and mesenchymal cells from the ocular tissue as well as from various sources have demonstrated their effectiveness in dampening neovascularization, scarring, and inflammation, while promoting epithelialization of the injured cornea. Moreover, a plethora of cytokines and growth factors, and extracellular vesicles, which constitute the secretome of these cells, work in concert to enhance wound healing. In this review, we provide an update on the recent potential therapeutic avenues and clinical applications of stem cells and their products in corneal regeneration after burn injury, as well as current imaging strategies for monitoring therapeutic efficacy and damage resolution.

## 1. Introduction

**Figure 1:**
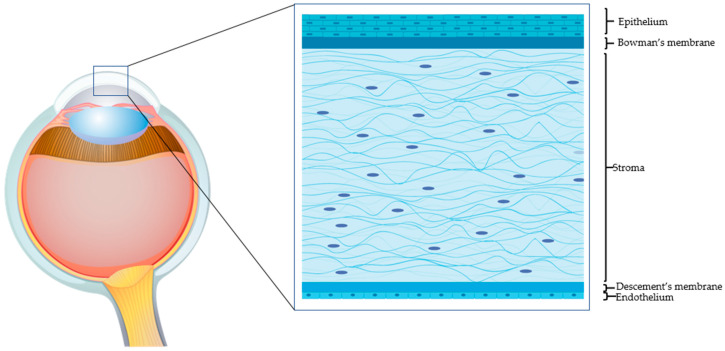
The human cornea. An epithelial cell layer is found on the outer surface of the cornea, followed by a basement membrane above Bowman’s layer. The middle stromal layer contains sparse keratocytes surrounded by strands of connective tissue called collagen fibrils. The Descemet’s membrane separates the stroma from the underlying endothelium, which is the innermost layer of the cornea.

The human adult cornea, though only five layers thick, performs crucial sensory and protective functions (Figure 1). The transparent tissue allows the transmission of light and, as a refractive lens, transmits light onto the light-sensitive retina. The cornea also protects the inner ocular tissue by acting as a physical barrier to the external insults. Any minimal alteration to the properties of the cornea (including refractivity or transparency) can affect its optical functions. The prevalence of corneal pathologies worldwide is very high, and ocular burns represent the major (88%) indication for surgery [1]. Morbidity from corneal injuries ranges from mild to potentially life threatening, hence impinging negatively on the quality of life and heavily on health care systems. The corneal epithelial cells have very high turnover (every 4 to 7 days) and allow the corneal surface to regenerate continuously [2]. The limbus, where the adult (epithelial and mesenchymal) stem cells reside, is the main source of corneal epithelial cells. A series of events contribute to the successful corneal epithelium turnover [3]. However, upon severe injury, when the endogenous regenerative capacity of corneal surface is impaired due to limbal damage, there is need for alternatives. Corneal transplantation is a last-option (requires trained personnel and sophisticated instruments and is expensive) and definitive solution. The shortage of corneal tissue donors, however, does not allow fulfilling all the requests for transplantation. With the recent biotechnological advances, it has been possible to devise innovative strategies to address the problems related to corneal injuries. Current surgical and pharmacological strategies for rescuing corneal functions host several exogenous factors and drugs. Growth factor-rich hemoderivatives that elicit corneal repair exploiting endogenous mechanisms have shown considerable promises [4]. Biomechanical modulation therapy is a new treatment modality, aiming at restoration of the limbal stem cell niche that is being exploited for its capacity to regenerate the cornea after burns [5]. Stem cells such as mesenchymal stromal/stem cells (MSCs) of different origin have also been employed in attempts to regenerate the damaged cornea. Coupled to 3D technology, these systems have offered tremendous advances in the field of corneal repair. Moreover, recent knowledge gained on the cell-free approaches, such as on extracellular vesicles (EVs), have offered numerous attractive possibilities in dampening corneal damage and in stimulating healing.

In this review, we provide an update on the recent potential therapeutic avenues and clinical applications of stem cells and their products in corneal regeneration after burn injury, as well as current imaging strategies for monitoring extent of resolution.

## 2. Corneal Burn Injury

**Figure 2:**
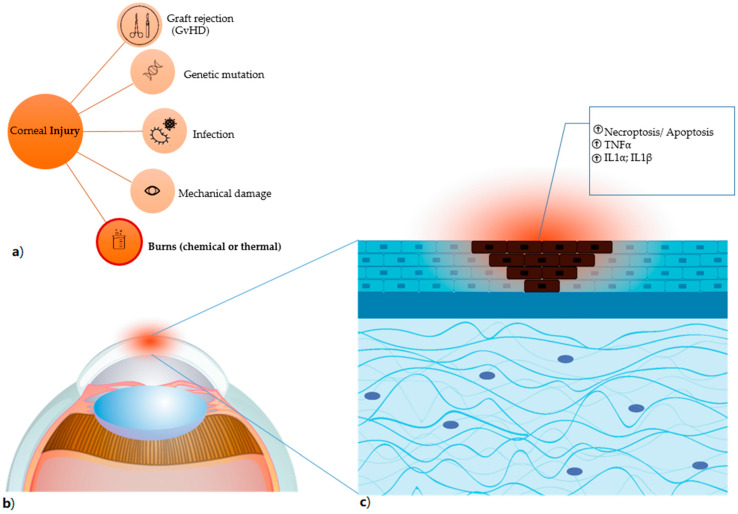
Corneal injury (**a**) the different types of corneal damages are listed, in particular burns, which comprise both chemical (for instance, induced by alkali exposure) and thermal burns. (**b**) Anatomical detail of the damaged corneal area, highlighted in red. (**c**) the damage caused by alkali burn induces necroptosis and apoptosis (shown as dark cells) with result in the release of factors that trigger the inflammatory process and have TNFα, IL1α and IL1β as primum movens.

Due to the close contact with the surrounding environment, the cornea is constantly at risk of being injured. Corneal injuries can be classified according to the type of trauma and the depth of the injury [6]. Corneal injuries comprise burns of various nature including chemical, thermal, radiation, and laceration (Figure 2). The resulting ocular trauma can be limited to the more anterior structures, such as the epithelium and stroma, or can penetrate deeper into the posterior structures such as the endothelium and anterior chamber up to the crystalline. Damage can be associated with hypoxia with consequent bacterial proliferation, hence necessitating ad-hoc treatments. Other causes of corneal degeneration are genetic and autoimmune such as type I diabetes.

Burns account for up to 18% of all reported ocular traumas [7]. Chemical corneal burns are very common in the workplace and are mainly caused by acidic and/or alkaline substances. Lipophilic alkalis have a much faster and deeper penetration capacity, and cause greater damage, with respect to acids [8,9]. Ensuing limbal stem pool deficiency may impair epithelialization, resulting in corneal neovascularization, lymph-angiogenesis, scar tissue formation, and immune response in the previously immune-privileged cornea. Secondary glaucoma may ensue, thus making it difficult to recover the corneal anatomical structure and physiological function, that is, sight [6,10]. 

The severity and depth of the damage, and the structures involved determine the outcome of corneal burns (Table 1). In fact, the deeper the damage is at the anatomical level and the more is the risk of infiltration by inflammatory cellular components. The latter induce, through release of cytokines and chemokines, remodeling of the stromal matrix, corneal neovascularization and lymph angiogenesis. Subsequently, scar-forming fibrotic processes entail, and blindness occurs.

## 3. Limiting Damage after Corneal Burn Injury

The management of chemical eye injuries can be classified according to the four phases of healing: immediate, acute, early reparative, and late reparative [11]. Management and minimization of damage after burn injury are the immediate aims of current strategies. Irrigation therapy is the most employed for immediate management, while anti-inflammatory therapies and agents for halting epithelial and stromal breakdown as well as for promoting corneal re-epithelialization and stromal healing are required for early reparative phases, which harness the endogenous repair mechanisms. Regarding the more severe ocular burns and chronic injuries, surgical (human amniotic membrane bandage, limbal tissue transplantation, and limbal stem cell transplantation) or pharmacological (autologous growth factors, and epigenetic regulation) approaches are evaluated [5]. Limbal stem cell transplantation is one of the most contemplated approaches to attempt corneal repair. Both endogenous and exogenous stem cell-based strategies, described below, are being developed, with some already in the clinical setting.

### 3.1. Endogenous Repair Mechanisms

#### 3.1.1. Cytokines and Chemokines

**Figure 3:**
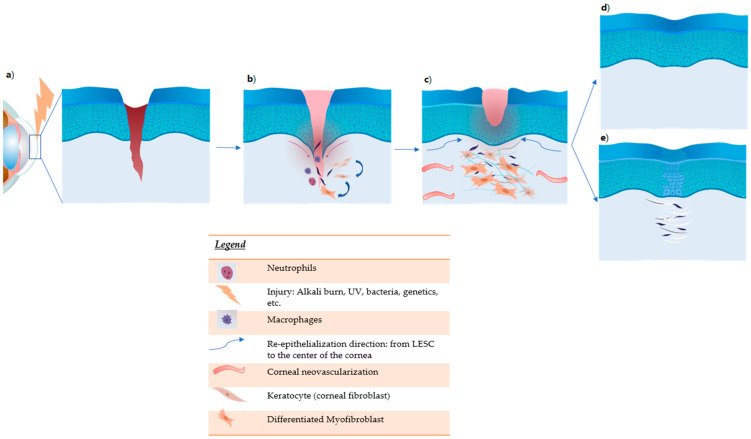
Corneal damage (deep injury) and wound healing. (**a**) Damage induces tissue necrosis and release of cytokines: TNFα, IL1, IL6, and PDGF. The acute phase of inflammation begins. (**b**) During inflammation, neutrophils and macrophages are recruited. They produce other inflammatory cytokines and chemokines. TGF-b induces the differentiation of fibroblasts into myofibroblasts. (**c**) Activation of limbal epithelial stem cells (LESC) proliferation. Remodeling of the stromal matrix due to MMP with vascular endothelial growth factor (VEGF)-induced vessel formation. (**d**) Treatment improves corneal damage with restoration of transparency. (**e**) In untreated cornea, tissue fibrosis with corneal opacity develops.

Upon burn damage, several primary biological mediators are released to elicit inflammatory response at the epithelial and stromal level. Tumor Necrosis Factor (TNF)-α is one of the first chemokines produced following necrosis of the epithelial tissue and induces the release of Interleukin (IL)-1, IL-6, IL-10, Transforming Growth Factor (TGF)-β and platelet-derived growth factor (PDGF), basic Fibroblast Growth Factor (bFGF), and vascular endothelial growth factor (VEGF) [12,13]. These factors guide competent inflammatory cells, such as monocytes, macrophages, and neutrophils, to the site of injury to start the process of wound healing: formation of new vessels and remodeling of the stromal matrix [14]. Neutrophils represent the most prominent inflammatory cells infiltrating the site of injury and are guided by the chemotaxis-assisting chemokine receptors (such as CCR1, CCR2, and CCR3). Neutrophils secrete pro-inflammatory cytokines and other inflammatory molecules, comprising IL-1β, IFN-γ, IL-17, and MMP-9 following alkali burn injury of the cornea [15]. IL-1β is a main pro-inflammatory molecule also produced by activated monocytes, macrophages, and dendritic cells. Some of these soluble factors were also found to be expressed in tears upon analysis of bilateral tear fluid protein levels during the wound healing process after corneal endothelial keratoplasty [16]. Insights into other cytokines and chemokines involved in the corneal repair from injury came from a previous large-scale microarray analysis on cultured mouse corneal stromal cells [17]. In particular, during the injury process, the expression of several corneal transparency-related genes (such as crystallins) was down-regulated while that of acute phase response genes (such as Saa3) and pro-inflammatory genes (such as Ccl2, Ccl7, and Ccl9) was induced. Upon repair, inflammatory cytokine genes (such as IL6st, Ccl7) and ECM remodeling genes (such as MMP3, MMP12) expression was repressed while profibrogenic genes (like Col1a1, Col3a1) and interstitial ECM synthesis genes (for instance, collagen types 1, III, V, and fibronectin) expression was up-regulated [17]. The matrix metalloproteinase (MMP) family of proteinases are crucial for corneal wound repair [18]. The expression of several MMPs, such as MMP-2, MMP-12, and MT1-MMP, is induced by cytokines to regulate wound repair [19,20] (Figure 3). The normal anti-angiogenic conditions of the resting cornea are also disturbed. Corneal avascularity is regulated by the endogenous anti-angiogenic factors such as VEGF receptor 3, soluble fms-like tyrosine kinase-1 (sFLT-1 or soluble VEGF receptor 1), and pigment epithelium derived factor (PEDF) [21].

#### 3.1.2. Endogenous Stem Cells

The capacity to replace damaged cells and repair tissues is one of the most wonderful tasks of endogenous stem cells. Corneal burn injury can be resolved mainly by two types of limbal stem cells: limbal epithelial stem cells (LESCs) and the recently discovered limbal mesenchymal stromal/stem cells (LMSCs). LSECs migrate from the limbus onwards towards the cornea to replenish the damaged area with new cells. Different approaches have been adopted using LSEC transplantation (LSCT) to achieving the regeneration of a stable, epithelialized as well as avascular corneal surface (reviewed elsewhere [1]). Shanbhag et al. recently analyzed, in a systematic review, all autologous approaches with LSECs and found these to be safe with an overall functional restoration rate of 60% and anatomical repair rate of 69% [1]. Another population of cells with MSC characteristics (LMSCs) exists in the corneal stroma and limbus [22]. These cells localize sub-adjacent to the limbal basement membrane and were found to enhance the viability as well as potency of the LESCs [2]. Importantly, LMSCs show multipotent features comprising differentiation into keratocytes, and perform essential corneal duties such as production of normal corneal extracellular matrix components including collagens. These cells are also capable of regulating corneal stroma remodeling and restore normal corneal transparency [23]. LMSCs can also regulate inflammatory and angiogenic processes [21]. LMSCs have great potential in the field of corneal burns, and understanding how these cells can be modulated endogenously, when the limbus is intact, to more efficiently direct tissue repair will be a great step forward. Corneal stromal stem cells (CSSCs) derived from the central part of the corneal stroma have also been isolated and characterized [24]. These CSSCs express mesenchymal and stemness-related surface markers characteristic of MSCs, and are capable of regulating corneal homeostasis, as well as regenerating the cornea following tissue injury [25,26]. Hence, the term corneal stromal MSCs (CSMSCs) or corneal MSCs (co-MSCs) has been coined for the CSSCs, which add up to the repertoire of endogenous cells that can repair the cornea upon injury [21].

### 3.2. Exogenous Restoration Strategies

#### 3.2.1. MSCs as Example of Stem Cell-Based Approach

MSCs have been historically described as “stem cells” but due to lack of data supporting their stemness, these cells are now cautiously referred to as “stromal cells”. In fact, MSCs isolated from different tissues are a heterogeneous population of cells, which may contain a subset of mesenchymal cells with stem cells activity [27,28,29]. MSCs are fibroblast-like cells which show particular characteristics such as adhesion to plastic, expression of surface markers, such as CD90, CD105, CD73, and CD29, and lack of CD34, CD45, and CD79, and multipotency for the possibility to induce these cells to differentiate into osteoblasts, adipocytes and chondroblasts in vitro [30]. As adult cells, MSCs are genetically stable with respect to induced pluripotent stem cells (iPSCs) and are subject to fewer ethical concerns compared to embryonic stem cells [31]. MSCs are easily harvested from a large variety of human tissues and cultivated in vitro. Differences in phenotypic markers, as well as proliferation capability has been observed among MSCs isolated from various sources, as for example, MSCs derived from bone marrow and adipose tissue differentially express CD34, CD49d, CD54 and CD106 [32,33]. MSCs injected in vivo by different routes can modulate immune response and induce repair of injured tissue not only by transdifferentiating but also by their paracrine action on cells, such as release of inflammatory cytokines and growth factors as well as EVs (described below) [34]. For instance, in a murine model of corneal alkali-burn, intravenously-injected bone marrow-derived MSCs efficiently migrated and engrafted in the wounded cornea possibly assisted by the action of stromal cell-derived factor (SDF)-1 and substance P [35,36]. These cells possess tissue protective and regenerative attributes together with immunomodulatory, anti-inflammatory, pro-angiogenic, and anti-fibrotic capacities, and have shown promises in corneal regeneration (recently reviewed in [37]). In a preclinical model, intrastromal transplantation of human umbilical cord MSCs also improved transparency of corneas injured by lamellar keratectomy [38]. Improvement in corneal opacity was also shown after injection of adult human corneal stem cells in mice with scar-like disruption of corneal transparency caused by lumican deficiency [24]. Subconjunctival injection of bone marrow-derived MSCs accelerated the wound healing process, attenuated inflammation and caused a reduction in neovascularization in corneal alkali burn rat models [39]. Interestingly, human MSCs isolated from healthy donors were grown and expanded on amniotic membrane and successfully used for reconstruction of rat corneas after chemical burns [40].

Conditioned media (CM) from adipose tissue-derived MSCs have also been recently reported as potential ophthalmic eye drop due to their growth factor-rich content [41]. Interestingly, the cell-free CM enhanced corneal regeneration in a chemical burn model. CM contain several corneal repair-inducing mediators. For instance, CM from bone marrow-derived MSCs were characterized and found to be rich in keratinocyte growth factor (KGF), TGFβ, PDGF, VEGF, bFGF, epidermal growth factor (EGF), and hepatocyte growth factor (HGF) [41]. Importantly, corneal MSCs also have anti-angiogenic properties and contribute to the corneal avascularity through the production of factors such as sFlt-1 and PEDF [21]. This was shown in vitro by the inhibition of vessel sprouting and endothelial tube formation upon treatment of human umbilical vein endothelial cells (HUVECs) with supernatant derived from cultured human corneal MSCs. In vivo, in a mouse model of corneal debridement, application of MSCs embedded in fibrin gel effectively prevented neovascularization [21]. Moreover, in mice, when applied to corneal wounds embedded in fibrinogen, which gelled in response to thrombin, human limbal biopsy-derived stromal cells promoted regeneration of native stromal tissue, and reduced neovascularization during the wound repair process [42].The effect of MSCs on wound closure was also investigated by applying MSC secretome in hyaluronic acid/ chondroitin sulphate (HA/CS) gel carrier on corneal wounds in vivo. Mice treated with MSC secretome had accelerated wound closure and absence of sub-epithelial scarring and fibrosis with respect to saline control groups [43]. However, despite advances in MSC research, the clinical application of these cells in the ophthalmic setting is still a concern, especially after adipose tissue-derived MSC therapy in three patients with macular degeneration resulted in severe visual loss and ocular complications [44]. Thus, much hope has turned towards cell-free therapies.

#### 3.2.2. Cell-Free Strategies: EV-Based Therapeutic Approaches for Corneal Burn Injury

**Figure 4:**
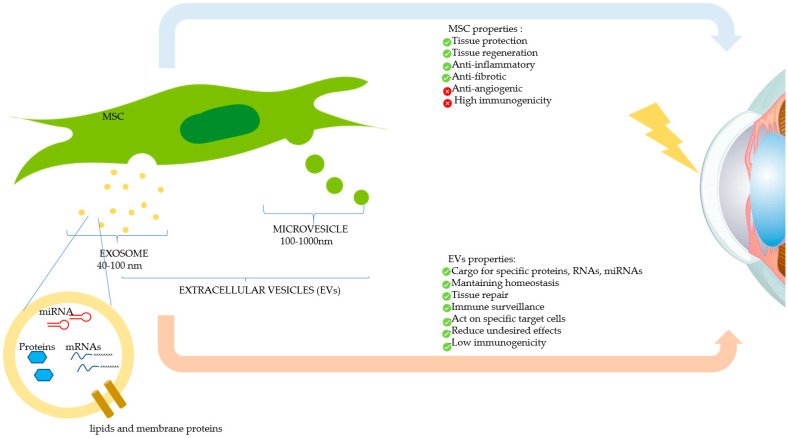
Therapeutic effects of mesenchymal stromal/stem cells (MSCs) and MSC-derived extracellular vesicles (EVs) on the damaged cornea. MSCs can modulate corneal repair through its paracrine action, by secreting soluble factors, or by releasing EVs, which contain therapeutic biomolecules. The two main types of EVs (exosomes and microvesicles) are shown.

Recently, research on EVs has gained tremendous ground in the field of regenerative medicine. EVs are released by virtually all cell types of the body and are frequently classified into heterogeneous EV subsets as exosomes, microvesicles, or apoptotic bodies, depending on their biogenesis and biophysical properties (Figure 4) [45,46]. Due to their ubiquity and ease of propagation in vitro, MSCs are considered excellent generators of EVs for clinical use. This kind of cell-free therapy offers several advantages over cell therapy, as for Graft versus Host Disease (GvHD) due to EVs’ low immunogenicity, and for tissue regeneration and organ protection [47]. EVs play a relevant role in intercellular communication thanks to their capacity to transfer multifarious cargos including proteins, RNA and lipids, as well as other components of the cytosol enclosed in a lipid bilayer, hence shielding these biomolecules from degradative enzymes [48]. These biomolecules play an important role in maintaining tissue homeostasis such as stem cell maintenance, tissue repair, immune surveillance, and blood coagulation [49]. EVs are being increasingly recognized for their wide-ranging therapeutic efficacy and biomarker potential, and as innovative drug delivery systems. EVs from different sources have been investigated for their therapeutic effects in different animal models of human diseases [50]. Further studies have shown that EV-based drug delivery systems may provide unique advantages, such as efficient targeting of their cargo into cells and reduced undesired effects due to their capacity to act on specific target cells [51]. Interestingly, EVs derived from CSSCs were shown to reduce inflammation, scarring, and fibrosis, hence improving corneal transparency, in a mouse model of corneal debridement [52]. The reparative effect was caused by the microRNA (miRNA) content of the EVs, as demonstrated by the lack of corneal wound healing upon knock-down of the ESCRT protein, Alix, required for miRNA transfer into exosomes. EVs, due to their biological nature and contents, are believed to be more efficient than liposomes bearing the same amount of biomolecules [51]. Topical instillation of EVs can be envisaged in the case of corneal burn injury. This can be facilitated by use of polysaccharides, such as methylcellulose, which may act as potential vehicles for prolonging EV residence time on the injured surface. Encouragingly, two clinical trials using cell-derived EVs in ocular diseases are on www.clinicaltrials.gov website (Table 2); to our knowledge, the use of EVs on corneal burns is still in the preclinical stage. In summary, EVs represent an innovative and very promising drug delivery system, and EV-based therapeutics offer new hopes to satisfy unmet clinical needs.

#### 3.2.3. Cell-Free Strategies: miRNAs as Valid Target in Corneal Burn Injury

MiRNAs, a subclass of 18–24 bp long non-coding single-stranded RNA, are an important regulatory RNA component of EVs. MiRNAs regulate stability as well as post-transcriptional regulation of gene expression by binding to target messenger RNAs (mRNAs) [54]. These biomolecules participate in various diseases including chronic inflammatory diseases, cancer, heart diseases, and ophthalmic diseases. In the case of corneal injury, the role of miRNAs on phenomena such as wound repair, inflammation, maintenance of the corneal/ limbal stem cell pool, lymphangiogenesis, and corneal neovascularization has been studied in the pre-clinical setting. For instance, in a rat model of alkali-induced burn injury, the authors evaluated the therapeutic effect of bone marrow-derived MSCs engineered to over-express miR146a on corneal repair [54]. A reduction in corneal opacification was observed in vivo with improvement in angiogenesis through the inhibition of VEGF, CD45 and Interferon-γ [54]. RNA-seq and qualitative proteomics analyses revealed that miR146a was upregulated in the limbal region rich in stem cells. The authors identified 251 mRNA targets of miR146a, in particular, notch1 (up-regulated) and notch2 (down-regulated), anchoring junctions, TGFβ, mTOC2, TNF-α and EGF receptor, which are involved in inflammation and maintenance of the correct stem pool [55]. Several other miRNAs have been reported to be involved in the angiogenic pathway, such as miR31 that is capable of regulating hypoxia inducible factor hence conferring an anti-angiogenic effect [56].

Regarding wound repair, miR145 was found to have an important role in the differentiation and function of myofibroblasts through regulation of the α-Sma (pro-fibrotic gene) and Klf4 (stem cell gene), in a model of UV-induced lesion [57]. Another miRNA, miR199a/b-5, was shown to down-regulate the expression of Discoidin domain receptor (DDR)-1, involved in lymphangiogenesis, in a rat alkali injury model [58]. Thus, these examples show that regulation of miRNA levels is crucial and that miRNAs can become a valid target for regulating and promoting wound healing in corneal burn injury.

## 4. Current Imaging Strategies to Evaluate Corneal Injury Resolution

While several vital stains such as sodium fluorescein, rose Bengal and lissamine green B are commonly used to assess ocular complications in the clinical setting, there are several limitations regarding their proper use and diagnostic utility [59]. Lately, non-invasive and non-destructive molecular imaging technologies have gained ground not only for the evaluation of ocular damage, but also in the monitoring of cell-based products applications as therapeutic intervention in the ocular tissue.

### 4.1. Molecular Imaging Approaches in the Assessment of Corneal Damage

**Figure 5:**
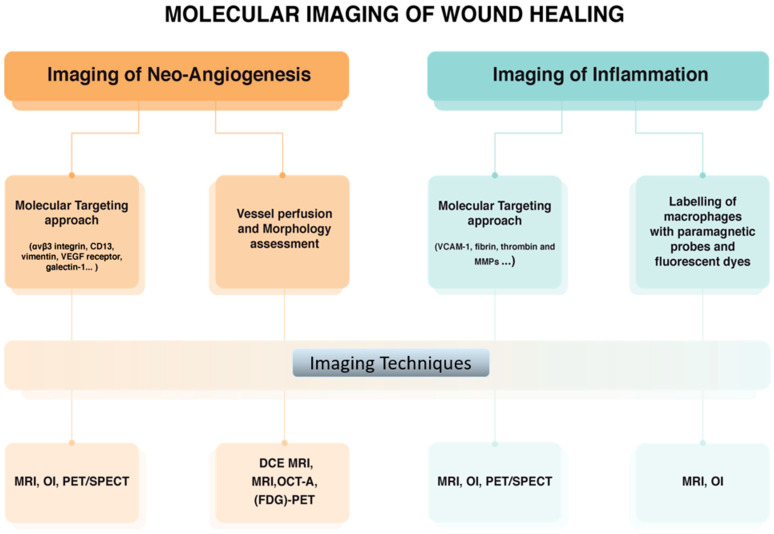
Molecular imaging approaches used for monitoring corneal wound healing. (MRI: magnetic resonance imaging, PET: positron emission tomography, SPECT: single photon emission computed tomography, CT: ultrasound imaging and computed tomography, DCE: Dynamic Contrast Enhanced, FDG: ^18^F-Fluorodeoxyglucose, OI: Optical imaging, OCT-A: Optical coherence tomography angiography).

Corneal burn results in wounding and, as a consequence, neo-angiogenesis, lymphangiogenesis, and scar develop during the healing process as described above. Diverse molecular imaging approaches have been employed for monitoring wound healing in vivo (Figure 5). Imaging of angiogenesis in diseased tissue essentially exploits vascular targeting. For this purpose, several targets that are upregulated in newly formed blood vessels have been identified through genomic screening approaches, and probes binding selectively to these targets have been developed. The main angiogenesis-related endothelial cell markers are αvβ3 integrin, VEGF receptor, CD13, vimentin, and galectin-1 [60,61]. Different imaging techniques have been successfully applied in preclinical studies of angiogenesis such as Magnetic Resonance Imaging (MRI), Positron Emission Tomography (PET), Single Photon Emission Computed Tomography (SPECT), Ultrasound Imaging (US), and Computed Tomography (CT) [62]. So far, Dynamic Contrast Enhanced (DCE) MRI represents one of most promising approaches especially to study tumor angiogenesis in cancer [63]. More recently, MRI, CT and ^18^F-Fluorodeoxyglucose (FDG)-PET studies of angiogenesis have been conducted to assess vessel permeability and density in atherosclerosis [64,65]. As mentioned above, αvβ3 integrin is a significant example of endothelial cell marker, being the binding target for several peptides based on the RGD (Arg-Gly-Asp) tripeptide. A wide variety of radio-labelled RGD analogues has been developed for PET and SPECT imaging [66]. Very often, these specific contrast agents are carried by biocompatible nanosystems capable of delivering their payload to the site of interest in order to overcome the sensitivity issues due to the very low in vivo concentration of such markers [67].

**Figure 6:**
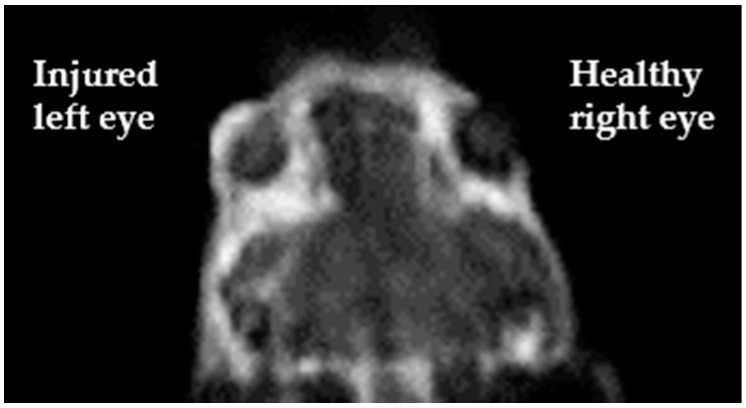
T_1_-weighted image, acquired using Gd-AAZTA-MADEC at a 1T spectrometer, clearly displays a brighter signal on the corneal surface of the alkali-exposed injured eye (**left**) with respect to the untreated one (**right**) (pers. comm.).

Regarding Magnetic Resonance Angiography (MRA), one approach regards the use of contrast agents that remain in the vascular space. The vast majority of T_1_-based contrast agents approved for clinical use consists of aspecific small molecules able to extravasate in the extracellular medium, so they are not suitable for MRA purpose. Two main strategies have been adopted to develop the so-called Blood Pool Agents (BPAs) for MRA: (i) to design molecules capable of high affinity binding to serum proteins (albumin is the best choice among serum proteins since it is the most abundant; non-covalent bonds are preferred for toxicity reasons); (ii) to use very large molecules the size of which prevents their extravasation. Clinically approved BPAs are either Gd-DTPA complexes coupled with moieties designed to reversibly bind serum albumin or larger-sized agents [68,69]. It is worth mentioning a novel T_1_ MRI blood-pool contrast agent (Gd-AAZTA-MADEC) that has shown, in preclinical studies, a drastic increase in the binding affinity towards mouse and human serum albumins and peculiar pharmacokinetics and relaxometric properties, making it a very promising contrast agent in angiography and tumor vascular microenvironment [70]. In a current study by our group, Gd-AAZTA-MADEC has been successfully used as MRI contrast agent to visualize the formation of new vessels occurring two days after the corneal injury in a murine model of corneal alkali burn (Figure 6, pers. comm.).

A vast number of studies on imaging of inflammation has been reported, using the main aforementioned imaging methodologies. Whenever the inflammation is associated with the presence of oedema, standard imaging techniques can visualize the inflamed regions. Otherwise, two main molecular imaging approaches have been reported consisting in targeting of specific inflammation markers, such as VCAM-1, fibrin, thrombin and MMPs [60,71] and ex vivo labelling of macrophages [72,73]. Recent studies have also revealed the role of non-contact imaging modalities for limbal injury and corneal wound healing [74,75]. Among these, a very promising approach to image the vasculature in the eye districts is represented by Optical Coherence Tomography Angiography (OCT-A), a technology based on laser light reflectance of moving red blood cells to obtain high resolution images of surface vessels without using exogenous dyes. Recently, Patel et al. showed that OCT-A can reliably and accurately detect as well as quantify relative changes in the perilimbal vasculature, hence distinguishing between healthy eyes and eyes with pterygium [74].Taken together, the results of these studies are very promising, even though none of these strategies have yet been translated to the clinic.

### 4.2. Molecular Imaging Approaches in the Monitoring of EV-Based Therapeutic Efficiency

In vivo tracking of stem cells using imaging approaches employs different techniques such as optical imaging (OI) (Fluorescence Imaging (FLI) and Bioluminescence Imaging (BLI)), PET, SPECT, CT, Photoacoustic Imaging (PAI), and MRI, and is mainly at the preclinical level. For instance, gold nanosphere-labelled MSCs have been injected ex vivo through the cornea into the anterior chamber of porcine eyes, and efficiently monitored by a ultrasound/PAI platform [76]. On the other hand, in vivo imaging of EVs applied for corneal regeneration would be decisive in understanding their therapeutic potential. Unfortunately, very few studies exist on in vivo EV imaging of the cornea. However, promising results have been reported so far on EV labelling and tracking with different methodological strategies, the translation of which to corneal imaging could be envisaged.

For all the above-mentioned modalities, EV labelling with a proper contrast agent is necessary for their in vivo tracing. Grange et al. compared direct and indirect labelling procedures in MSC-derived EVs used as therapeutics in mice with kidney injury, and found that both techniques are suitable for FLI-mediated in vivo tracking in the first 5 h after injection [77]. Labelled EVs isolated from different human breast cancer cells have been successfully imaged to assess their biodistribution [78]. Thus, FLI, despite the low penetration disadvantage, is a powerful technique to track EVs and to elucidate their main mechanism of action. Among the other imaging modalities used for tracking and studying the bio-distribution of EVs in vivo, nuclear techniques such as PET and SPECT play an important role. The labelling of the EVs can be done by direct incubation of the EVs with radionuclides. For instance, Smyth et al. labelled EVs with ^111^In to measure the biodistribution of EVs ex vivo [79]. This experiment revealed a fast clearance of the EVs from the blood in PC3 tumor bearing mice. Hwang et al. used ^99^Tc to detect the biodistribution of labelled EVs directly in vivo [80]. They measured a high signal coming from the liver even 5 h post injection. Nuclear imaging techniques have very high sensitivity that is a mandatory requisite to detect low concentrations of EVs. CT and PAI have been also used to label EVs for in vivo tracking, with promising results [81,82]. Regarding the visualization of EVs by MRI, several studies were performed with Super Paramagnetic Iron Oxide nanoparticles (SPIO) as contrast agents trapped inside the EVs. Given the large dimensions of these magnetic probes, electroporation was used for their internalization into EVs. Hu et al. successfully traced the in vivo fate of SPIO-labelled EVs injected into the foot pad, and found that EVs preferentially accumulate in some very specific areas of the lymph nodes [83]. Smaller oxide particles such as USPIO (Ultra Small Paramagnetic Iron Oxide nanoparticles) were used by Busato et al. to label EVs indirectly before their extraction from adipose tissue [84]. Even though SPIO and USPIO are very sensitive, the risks associated with their use reside in the possibility that their long-term in vivo fate differs from that of the EVs [84,85]. This latter drawback occurs for all techniques that need direct labelling since, in the long-term, the contrast agent contained in the vesicles could be lost or transferred so that the acquired signal can no longer be attributed with certainty to EVs.

As a step forward, multimodal approaches capable of combining the specific advantages of the different imaging techniques to image and track EVs are increasingly being devised. For instance, Shaikh et al. described a study on tumor diagnosis with EVs using three different imaging modalities, such as CT, FLI, and MRI [86]. Thus, strategies for EV labelling and their in vivo tracking, albeit in their early days, are indeed very promising.

## 5. Concluding Remarks

**Figure 7:**
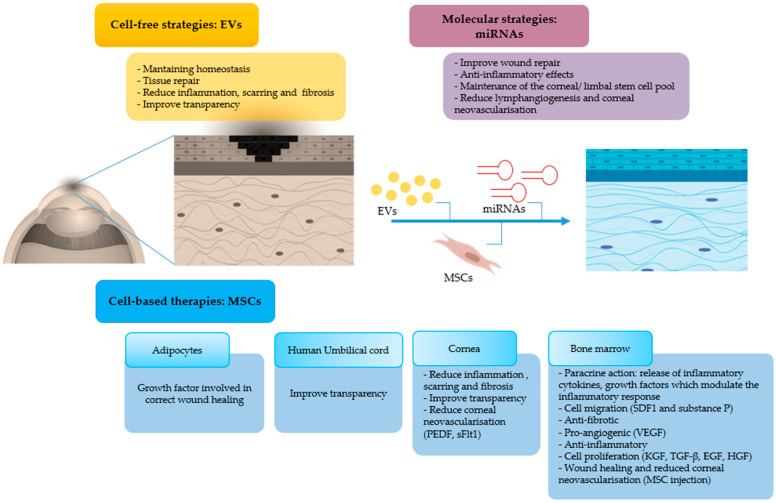
Mechanism of action of MSCs and their derivatives, in particular EVs and miRNAs, on the resolution of corneal burns (MSCs: mesenchymal stem cells, EVs: extracellular vesicles, miRNAs: microRNAs, PEDF: pigment epithelium derived factor, sFlt-1 soluble fms-like tyrosine kinase-1, VEGF: vascular endothelial growth factor, TGF: transforming growth factor, EGF: epidermal growth factor, HGF: hepatocyte growth factor, KGF: keratinocyte growth factor, SDF: stromal cell-derived factor).

MSC-based therapy have given encouraging results for corneal repair after burn injury, and the levels at which MSCs and cell-free strategies mechanistically impact on wound healing after corneal burns are depicted in Figure 7. Several aspects however need improvement. For instance, dosage and route of EV application need to be optimized in order to obtain the desired therapeutic effects whilst avoiding side effects on the long-term. The optimal labelling of EVs for their successful in vivo tracking in the cornea is another issue that requires further studies. IPSCs have also been proposed for the treatment of ocular diseases. It was recently reported that sheets of corneal cells derived from iPSCs were transplanted in one patient in order to restore vision and that the procedure has been approved in three other patients [87]. This increases the list of stem cells that could be employed for corneal regeneration after burn injury and is a crucial step forward in the field.

## Figures and Tables

**Table 1:** Severity and depth of corneal damage and grading.

Degree of Chemical Injury	Aim	Clinical Treatment	Follow-up
Grades I and II	Reduce the exposure of the chemical agent by decreasing its concentration and restoring the pH of the tear film and the surface involved	- Sterile saline buffered with HCO_3_^−^ - Lactated ringer’s solution - Balanced sterile saline solution - Glucose solution (lesion given by lime)	This damage has a good prognosis and must be treated with antibiotics and artificial tears.
Grades III and IV	The debridement of the necrotic tissue reduces inflammation, promoting re-epithelialization. In general, it is important to intervene in the first week after the injury to have a quick recovery and to avoid blindness.	- Debridement of the necrotic epithelium - Limbal stem cell transplant - Cultured oral mucosal epithelium transplant (COMET) - Amniotic Membrane transplant (AMT)	If proper follow-up is not done, dry eye and secondary glaucoma can occur

**Table 2:** Current clinical trials employing EVs in ocular diseases [53]; MSC: Mesenchymal stem cells; cGVHD: corneal Graft versus Host Disease.

Nct Number	Title	Type of Eye Disease	Source of EVs	Regimen/Application Route	Status/Objectives	Study Start Date	Study Type/Country
NCT04213248	Effect of UMSCs- Derived Exosomes on Dry Eye in Patients With cGVHD	Dry Eye in Patients With cGVHD	Umbilical Mesenchymal Stem Cells (UMSCs)	Artificial tears for 2 weeks followed by UMSC-derived exosomes 10ug/drop, four times a day for 2 weeks	Recruiting/assess the alleviation of dry eye symptoms in patients with cGVHD; measure tear secretion	February 18, 2020	Phase 1-Phase 2/China
NCT03437759	MSC-Exosomes Promote Healing of Macular Holes	Macular Holes	Human umbilical cord mesenchymal stem cells	Intravitreal injection; 50 μg or 20 μg MSC-Exosomes in 10 μL PBS	Recruiting/assess the safety and efficacy of MSC-derived exosomes in promoting healing of large and refractory macular holes	March 1, 2017	Early Phase 1/China
